# MRAP deficiency impairs adrenal progenitor cell differentiation and gland zonation

**DOI:** 10.1096/fj.201701274RR

**Published:** 2018-06-07

**Authors:** Tatiana V. Novoselova, Mashal Hussain, Peter J. King, Leonardo Guasti, Louise A. Metherell, Marika Charalambous, Adrian J. L. Clark, Li F. Chan

**Affiliations:** * Centre for Endocrinology, William Harvey Research Institute, Barts and the London School of Medicine, Queen Mary University of London, London, United Kingdom;; † Medical and Molecular Genetics, King’s College London, London, United Kingdom

**Keywords:** ACTH, accessory protein, melanocortin, cell fate, familial glucocorticoid deficiency

## Abstract

Melanocortin 2 receptor accessory protein (MRAP) is a single transmembrane domain accessory protein and a critical component of the hypothamo-pituitary-adrenal axis. MRAP is highly expressed in the adrenal gland and is essential for adrenocorticotropin hormone (ACTH) receptor expression and function. Human loss-of-function mutations in *MRAP* cause familial glucocorticoid (GC) deficiency (FGD) type 2 (FGD2), whereby the adrenal gland fails to respond to ACTH and to produce cortisol. In this study, we generated *Mrap*-null mice to study the function of MRAP *in vivo*. We found that the vast majority of *Mrap*^−/−^ mice died at birth but could be rescued by administration of corticosterone to pregnant dams. Surviving *Mrap*^−/−^ mice developed isolated GC deficiency with normal mineralocorticoid and catecholamine production, recapitulating FGD2. The adrenal glands of adult *Mrap*^−/−^ mice were small, with grossly impaired adrenal capsular morphology and cortex zonation. Progenitor cell differentiation was significantly impaired, with dysregulation of WNT4/β-catenin and sonic hedgehog pathways. These data demonstrate the roles of MRAP in both steroidogenesis and the regulation of adrenal cortex zonation. This is the first mouse model of isolated GC deficiency and reveals the role of MRAP in adrenal progenitor cell regulation and cortex zonation.—Novoselova, T. V., Hussain, M., King, P. J., Guasti, L., Metherell, L. A., Charalambous, M., Clark, A. J. L., Chan, L. F. MRAP deficiency impairs adrenal progenitor cell differentiation and gland zonation.

Melanocortin receptor accessory proteins (MRAPs) are a class of single-pass transmembrane domain accessory protein (1–3). MRAP and MRAP2 have been shown to interact *in vitro* with the melanocortin receptors (MCRs), a family of GPCRs with diverse physiological functions that are stimulated by pro-opiomelanocortin–derived peptide agonists. MRAP2 is implicated in metabolic regulation *via* its interaction with MC4R, although other mechanisms may be involved (2, 4–7).

MRAP is essential for the function of the adrenocorticotropin hormone (ACTH) receptor/melanocortin 2 receptor (MC2R) and is highly expressed in adrenal glands (1). MRAP has been shown to form unique antiparallel homodimers that complex with, and assist, MC2R trafficking and signaling (8–10). The MRAP/MC2R complex on the surface of adrenocortical cells is essential to mediate the action of pituitary ACTH and subsequent steroidogenesis. Mutations in *MC2R* account for up to 25% of cases of familial glucocorticoid (GC) deficiency (FGD) type 1 (FGD1; OMIM:202200) (11, 12). FGD manifests in early childhood with hypoglycemia, lethargy, and overwhelming infection due to isolated GC deficiency while maintaining normal aldosterone levels. Mutations in *MRAP* are responsible for ∼20% of cases of FGD type 2 (FGD2; OMIM: 609196) and cause earlier disease onset compared with FGD1 (13).

Hormones secreted from the three concentric zones of the adrenal cortex are vital for life and general well-being. Mineralocorticoids (aldosterone) from the outer zona glomerulosa (ZG) regulate electrolyte levels and blood pressure. The inner zona fasciculata (ZF) is responsible for GC production (cortisol in humans and corticosterone in the majority of rodents, except the spiny mouse that produces cortisol) and is important for the stress response and for metabolic and immune function. The most medial zona reticularis (ZR) in humans secretes adrenal androgens that control adrenarche in puberty. Unlike in humans, the innermost zone in mice is called the X zone. This distinct zone is the remaining remnants of the fetal zone, which regresses during the first pregnancy in female mice and at puberty in male mice (14). The mechanisms controlling adrenal cortex zonation and cell renewal are under increasing scrutiny and have been implicated in adrenal diseases, tumorigenesis, and aging (15, 16). In particular, the adrenal capsule, a thin covering of cells adjacent to the ZG, and the subscapular region are thought to house the pool of adrenal stem/progenitor cells capable of dividing, migrating centripetally, and differentiating into mature steroid-producing cell types (15–17). A number of paracrine and endocrine factors have been implicated in adrenal zonation and renewal, including sonic hedgehog (SHH) signaling, WNT/β-catenin pathways, and, more recently, ACTH-mediated PKA activation (15, 18, 19). Our previous work, localizing MRAP to the undifferentiated zone of the rat adrenal gland, allowed us to question the importance of MRAP in adrenal cell differentiation and maintenance (20). In this study, we generated *Mrap^−/−^* mice to investigate the function of MRAP in FGD pathophysiology and its role in adrenocortical development and maintenance.

## MATERIALS AND METHODS

### Generation of *Mrap^−/−^* mice

**Figure 1:**
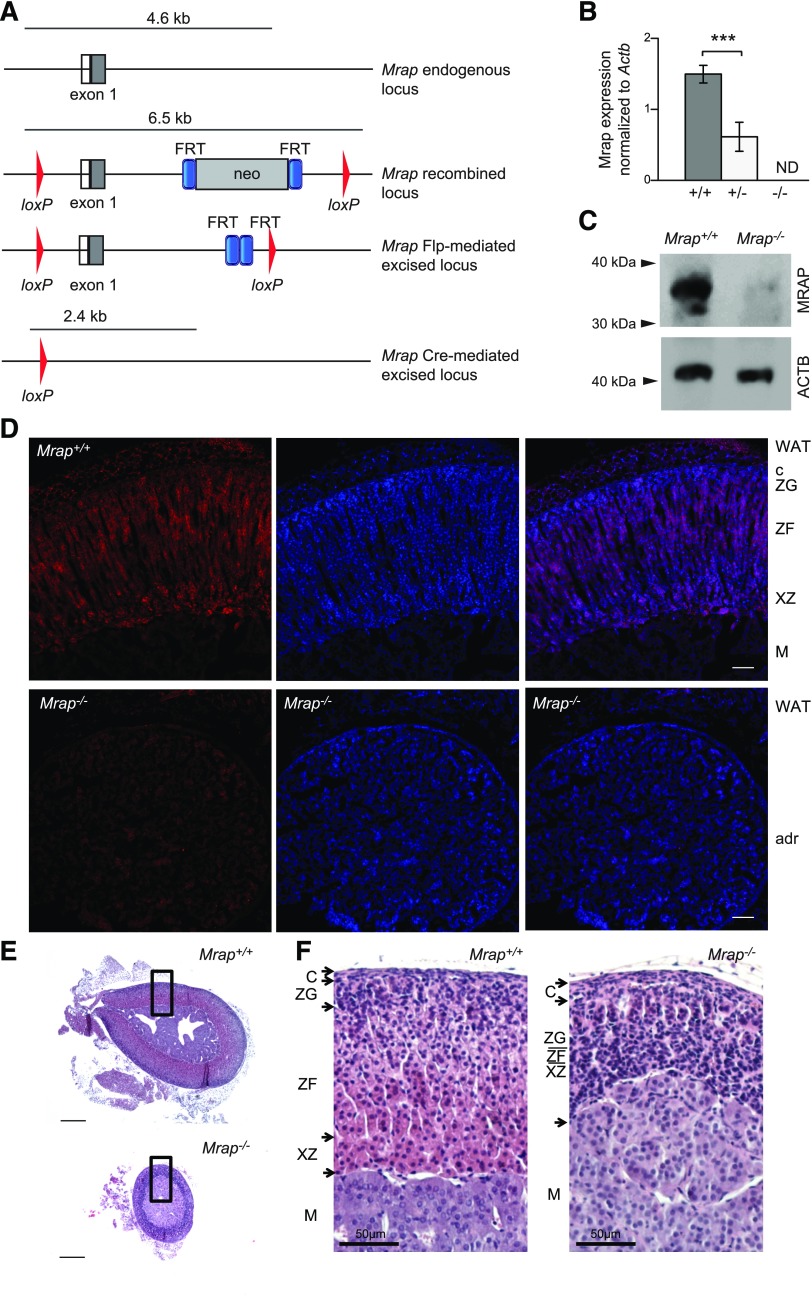
Generation of *Mrap^−/−^* mice. *A*) Schematic representation of targeting of the exon 1 of *Mrap* mouse for the line production. *B*) qRT-PCR analysis of Mrap expression in the adrenal gland of *Mrap^+/+^* (*n* = 7), *Mrap^+/−^* (*n* = 7), and *Mrap^−/−^* (*n* = 4) mice showing that *Mrap^+/−^* mice express approximately half of the *Mrap* transcript found in the wild-type adrenals, whereas no transcript was detected in *Mrap^−/−^*adrenals. ***P < 0.0005, Student’s *t*-test. *C*) Immunoblotting of adrenal gland lysates from *Mrap^+/+^* and *Mrap^−/−^* mice with anti-MRAP and anti–actin b (ACTB) antibodies. The arrows on the left indicate the approximate MW position. The position of the main band in *Mrap^+/+^* corresponds to the anticipated size of a mature MRAP dimer, with the lower band representing the nonglycosylated form. *D*) Immunostaining of MRAP (red) in the adrenal gland of *Mrap^+/+^* (top panel) and *Mrap^−/−^* mice (bottom panel). The nuclei are stained with DAPI (blue). The adrenal zonation is marked in the wild-type gland as follows: C, capsule; M, medulla; WAT, white adipose tissue; XZ, X-zone. Scale bars, 100 μm. *E*) Representative images of the adrenals from the *Mrap^+/+^* and *Mrap^−/−^* mice sectioned though the middle of the organ, demonstrating the reduction in the gland’s size. The black boxes indicate the where the higher-magnification images were obtained. Scale bars, 200 μm. *F*) Adrenal gland zonation is impaired in *Mrap^−/−^* mice (right panel) compared with *Mrap^+/+^* mice (left panel) as shown by the H&E staining. Panel images are from female mice, although similar adrenal gland zonation impairment is seen in male mice (data not shown). Adrenal gland zonation in male mice: C, capsule; M, medulla; XZ, X zone. The zonation in the *Mrap^−/−^* adrenal gland is not defined. Scale bars, 50 µm.

Design of targeting vector and generation of *Mrap^−/−^* mice was performed by Genoway (Lyon, France). The strategy is illustrated in **Fig. 1*A***. Briefly, the genomic region of interest was amplified by PCR and cloned into the targeting vector containing a neomycin selection cassette flanked by flippase recognition target sites and loxP flanking sites. All PCR fragments were validated by DNA sequencing. The targeting vector was electroporated into C57Bl/6 ES cells. G418-resistant clones were harvested and screened by PCR and Southern blot analysis. The recombined ES cell clones were microinjected into C57BL/6J blastocysts. To generate global *Mrap^−/−^* mice, male mice with the *Mrap* recombined locus were bred with female mice expressing Flp recombinase to remove the selectable marker. Animals were bred with germline Cre delete mice to excise exon 1 of *Mrap*.

### Animal husbandry, procedures, and hormone analysis

The care and use of all animals were carried out in accordance with the United Kingdom Home Office Regulations, United Kingdom Animals (Scientific Procedures). Mice were kept under standard 12-h light/dark cycle with food and water *ad libitum* unless stated otherwise. Corticosterone hormone replacement was performed by administering 50 μg/ml in drinking water to breeder cages starting embryonic day (E)17.5; drinking water was changed daily. The treatment was stopped at weaning [postnatal day (P)21] or continued until 8 wk of lifetime corticosterone replacement. An ACTH stimulation test was performed in the morning by intraperitoneal injection of 10 ng/g synacthen (Alliance Pharmaceutical, Chippenham, United Kingdom). Mice were euthanized by exsanguination 1 h after injection. For ACTH measurement, the animals were fasted for 16 h followed by exsanguination. Twenty-four–hour urine was collected using metabolic cages. Heparinized plasma was subject to ELISA for corticosterone and aldosterone (KGE009 and KGE016, respectively; R&D Systems, Abingdon, United Kingdom). ACTH was measured using a 6-Plex Luminex Assay (Thermo Fisher Scientific, Cheshire, United Kingdom). Urine catecholamines and creatinine were assessed by HPLC.

### Histology and histochemistry

Tissues were fixed in ice-cold 4% paraformaldehyde in PBS followed by paraffin embedding for hematoxylin and eosin (H&E) staining. For immunohistochemistry, tissues were cryoprotected with 20% sucrose in PBS after fixation followed by cryosectioning and heat-mediated antigen retrieval with citric buffer (10 mM, pH 6.0) as previously described (18). The primary antibodies used in this study were rabbit anti-MRAP 1/20 (10); mouse anti-CYP11B1 and rabbit anti-CYP11B2, both 1/50 (kind gift of Dr. C. E. Gomez-Sanches, University of Mississippi, Jackson, MI, USA); rabbit anti-PNMT 1/250 (Abcam, Cambridge, United Kingdom); mouse anti–β-catenin 1/300 (MilliporeSigma, Cambridge, United Kingdom); rabbit anti-WNT4 1/20 (Abcam); mouse anti-StAR 1/400 (Abcam), rabbit anti-20αHSD (1/5000, kind gift from Y. Weinstein, Israel), rabbit anti-DAB2 1:200 (Santa Cruz Biotechnology, Dallas, TX, USA), and rabbit anti-LEF1 1/100 (Abcam). *In situ* hybridization was performed as described by Guasti *et al.* (21). The *Shh* sequence used for RNA probe synthesis was derived from adult mouse adrenal cDNA using the primers forward, 5′-ATGCTGCTGCTGCTGGCCAGATG-3′ and reverse, 5′-GGGCCCCGAGTCGTTGTGCGGCG-3′. The 849 bp fragment was then ligated into pGEM-Teasy (Promega, Madison, WI, USA) and confirmed by Sanger sequencing. T7 and SP6 (Roche, Basel, Switzerland) were used for *in vitro* translation to produce the riboprobe. Capsule counts were performed using at least eight H&E-stained sections cut approximately through the middle, and capsule thickness (μm) was measured using the average of at least three measurements for each section analyzed. The images were analyzed and processed using Leica LAS AF (Leica Microsystems, Wetzlar, Germany), Adobe Photoshop, and Adobe Illustrator (San Jose, CA, USA).

### Western blotting

Adrenal glands were lysed in 2× Laemmli sample buffer, sonicated for 10 s, and centrifuged at 13,000 *g* for 15 min. The samples were then denatured for 1 h at 37°C and loaded onto 4–12% Bis-Tris Novex Acrylamide Gel (Thermo Fisher Scientific). Western blotting was performed per standard protocol and immunostained using rabbit anti-MRAP antibody 1/500 and mouse anti-actin b 1/10,000 (Abcam), anti-rabbit Odyssey Li-Cor 800, and anti-mouse Odyssey Li-Cor 680 secondary antibodies and visualized using the Odyssey Fc Imaging System (Li-Cor Biosciences, Lincoln, NE, USA).

### Quantitative RT-PCR

The tissue was dissected and immediately placed in liquid nitrogen. RNA was then extracted using the RNAsy Kit (Qiagen, Hilden, Germany), and 1 μg of RNA was used for cDNA synthesis using SuperScript IV RT (Thermo Fisher Scientific). TaqMan predesigned assays (Thermo Fisher Scientific, listed in Supplemental Data, and Supplemental Table 1) were then performed according to the manufacturer’s protocol, including no template or reverse transcription control, and analyzed using the Δ*C_t_* method.

### Statistical analysis

All values were calculated as means ± sem. Comparisons of two groups were analyzed by Student’s *t* test. For more than 2 groups, 1-way ANOVA was performed followed by Bonferroni multiple comparison test. In all analyses, a 2-tailed probability of *P* < 0.05 was considered statistically significant.

## RESULTS

### Generation and production of *Mrap*^−/−^ mice

*Mrap*^−/−^ mice were produced by deletion of exon 1 of *Mrap* (Fig. 1*A*). No *Mrap* transcript was detected in the knock-out animals, whereas *Mrap*^+/−^ mice produced approximately half of the transcript level found in the wild-type littermates (Fig. 1*B*). The lack of protein expression in *Mrap*^−/−^ mice was confirmed using an anti-MRAP antibody. Immunoblotting demonstrated a stable homodimer of MRAP, with electrophoretic mobility of ∼35 kDa in the adrenal gland of *Mrap*^+/+^ mice but not in the mutant (Fig. 1*C*). Immunohistochemistry showed that MRAP is detected mostly in the ZF in the wild-type adrenal gland with some staining in the X zone (Fig. 1*D* and Supplemental Fig. 1). This staining was not observed in the adrenal gland of *Mrap*^−/−^ mice, consistent with the absence of MRAP protein production (Fig. 1*D*).

### *Mrap* deficiency was associated with neonatal lethality, which was prevented with corticosterone replacement

Of the 325 mice generated from *Mrap^+/−^* intercrosses, only three *Mrap^−/−^* mice survived until weaning without corticosterone replacement. *Mrap^−/−^* mice were usually found dead before P0.5 without milk in their stomach. Excised lungs from deceased neonatal *Mrap*^−/−^ mice sank when submerged in water/saline, suggesting a failure of lung inflation. It is known that fetal GCs play an important role in fetal preparation for neonatal life by facilitating lung maturation and hepatic glycogen accumulation. We examined E19.5 embryos and found that the lungs of the mutant mice resembled immature lungs prior to terminal differentiation of the alveoli (Supplemental Fig. 2*A*). *Mrap* mRNA was not detected in wild-type fetal (at E15.5; Supplemental Fig. S2*B*) or adult lungs (Supplemental Fig. 2*C*) by quantitative RT-PCR (qRT-PCR), and fetal lungs stained negatively for *Mrap* expression by *in situ* hybridization at E14.5 (Supplemental Fig. 2*D*). Glycogen storage in the liver of *Mrap*^−/−^ E19.5 preterm embryos was reduced compared with *Mrap^+/+^* littermate controls (Supplemental Fig. 2*E*). These findings suggested that the pups probably died of failure to breathe and/or defective neonatal nutritional adaptation due to GC deficiency. Therefore, to produce *Mrap*^−/−^ mice and matched *Mrap^+/+^* littermate controls, we intercrossed *Mrap^+/−^* mice and administered corticosterone in the drinking water (50 μg/ml) of pregnant dams from E17.5 until weaning of the pups on P21. With this strategy, the survival of *Mrap*^−/−^ mice at weaning was close to the expected rate [61 (24.7%) *Mrap^−/−^*, 115 (46.7%) *Mrap^+/−^*, and 70 (28%) *Mrap^+/+^*]. *Mrap^−/−^* mice were visually indistinguishable from their wild-type, sex-matched littermates who had also received corticosterone from E17.5 until weaning. After weaning, the mice no longer received corticosterone in the drinking water. No adult mortality was noted in the surviving *Mrap^−/−^* mice, and both female and male *Mrap*^−/−^ mice were fertile.

### Surviving *Mrap*^−/−^ mice develop isolated GC deficiency

**Figure 2:**
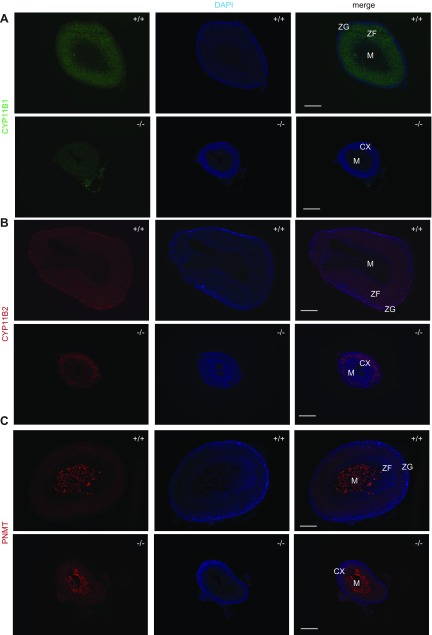
Impaired zonation in the adrenal cortex of the *Mrap^−/−^* mice. *A*) Severely reduced immunostaining of CYP11B1 (green) in *Mrap^−/−^* adrenal glands. *B*) CYP11B2 (red) localization in *Mrap^+/+^* and *Mrap^−/−^* adrenal glands. *C*) PNMT (red) in the adrenal medulla of *Mrap^+/+^* and *Mrap^−/−^* mice. The cell nuclei are stained with DAPI (blue). CX, cortex; M, medulla. Scale bars, 200 µm.

Adrenal glands in *Mrap*^−/−^ mice were macroscopically detectable but significantly reduced in size at 8 wk (Fig. 1*E*). This is in contrast to the relatively similar adrenal gland sizes between fetal *Mrap^−/−^* and *Mrap^+/+^* mice at E19.5 (Supplemental Fig. 3). Histologic analysis of adult adrenal glands revealed grossly abnormal zonation of the adrenal cortex in both male and female *Mrap^−/−^* mice (Fig. 1*F*). Immunohistochemistry demonstrated that the adrenal glands in *Mrap*^−/−^ mice were negative for CYP11B1, the key terminal enzyme essential for corticosterone production, suggesting the absence of GC-producing cells (**Fig. 2*A***). Expression of CYP11B2, the key enzyme for aldosterone production, was restricted to the ZG in the wild-type mice, whereas in the *Mrap*^−/−^ adrenals CYP11B2 stained a larger proportion of cells, with positive cells seemingly taking over large parts of the cortex (Fig. 2*B*). The adrenal medulla appeared to be unaffected as assessed by PNMT staining (Fig. 2*C*).

**Figure 3:**
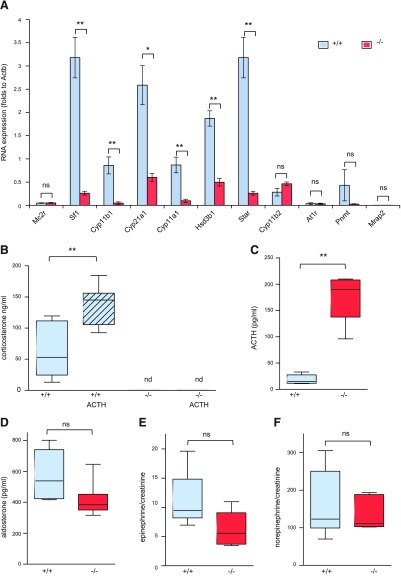
ACTH resistance and isolated GC deficiency of *Mrap^−/−^* mice. *A*) qRT-PCR of the adrenal gland of male *Mrap^+/+^* and *Mrap^−/−^* mice (*n* = 4 for each group) shows down-regulation of the steroidogenic pathway. *B*) Plasma corticosterone levels (ng/ml) at the basal level and in response to ACTH stimulation in male *Mrap^+/+^* (*n* = 7) and *Mrap^−/−^* (*n* = 7) mice. *C*) ACTH levels (pg/ml) in male *Mrap^+/+^* (*n* = 6) and *Mrap^−/−^* (*n* = 5) mice. *D*) Comparison of plasma aldosterone levels (pg/ml) between the genotypes in male mice (*Mrap^+/+^*, *n* = 7; *Mrap^−/−^*, *n* = 7). *E*, *F*) Epinephrine (*E*) and norepinephrine (*F*) levels in the 24-h urine of male *Mrap^+/+^* (*n* = 6) and *Mrap^−/−^* (*n* = 5) mice assessed as the ratio to creatinine to adjust for the kidney function. Student’s *t* test used for comparison of 2 groups. *P < 0.05, **P < 0.005, ***P < 0.0005. 1-way ANOVA for more than 2 groups. *P < 0.05, **P < 0.01, ***P < 0.001. NS, not significant.

Studying adrenal gland steroidogenic enzyme expression more broadly, RNA levels of *Cyp11a1*, *Hsd3b1*, *Cyp21a1*, and *Cyp11b1* as well as *Sf-1* and *Star* (steroidogenic acute regulatory protein) were significantly decreased in the adrenals of *Mrap*^−/−^ mice (**Fig. 3*A***) compared with wild-type, sex-matched littermates. The expression of the key enzyme of the aldosterone synthesis pathway, *Cyp11b2*, was not affected. These findings, together with histologic staining of CYP11B2-positive cells, suggest that this pathway is likely to remain functional. There were no changes of *Mc2r* expression in *Mrap*^−/−^ mice. Low *Mrap2* transcript levels detected in *Mrap*^+/+^ mice adrenals were unchanged in *Mrap*^−/−^ mice (Fig. 3*A*), indicating that MRAP’s only ortholog, *Mrap2*, is unlikely to compensate for the loss of *Mrap* expression at the RNA level. This is in keeping with previous *in vitro* data that much higher concentrations of *Mrap2* are required for the functional expression of MC2R (20).

To investigate the physiological effects of *Mrap* gene deletion, we examined the response of mutant mice to exogenously administered ACTH. We could not detect corticosterone in *Mrap*^−/−^ mice either basally or after ACTH stimulation, whereas ACTH response in wild-type mice was present in both genders (Fig. 3*B* and Supplemental Fig. 3*A*). ACTH response in both male and female *Mrap^+/−^* mice was intact and similar to *Mrap^+/+^* littermates (Supplemental Fig. 3*C*, *D*). The levels of plasma ACTH were greatly increased in *Mrap*^−/−^ mice compared with *Mrap*^+/+^ mice (Fig. 3*C*). Thus, *Mrap^−/−^* mice demonstrated ACTH resistance.

Aldosterone levels were unaffected in mutant mice of both genders (Fig. 3*D* and Supplemental Fig. 3*B*), as were urinary catecholamine concentrations (Fig. 3*E*, *F*). This *Mrap*^−/−^ mouse phenotype differs from *Mc2r^−/−^* mice, which have been reported to have partial aldosterone and catecholamine deficiency in addition to GC deficiency (22). The *Mrap*^−/−^ mouse therefore closely replicates the human FGD phenotype of isolated GC deficiency.

### *Mrap^−/−^* adrenal glands have a thickened capsular layer, which was partially rescued with GC treatment

**Figure 4:**
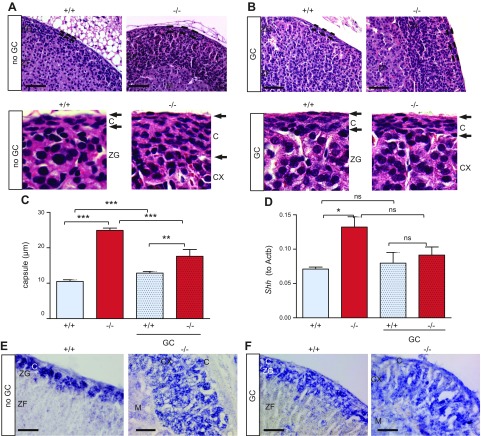
Capsular layer is thickened in *Mrap^−/−^* mice. *A*, *B*) Representative images of *Mrap^−/−^* adrenal glands and of wild-type littermates showing increased capsule thickness (*A*; highlighted with black bars) and in response to lifetime GC treatment (*B*). The bottom panels show digital magnification of the adrenal gland at the capsular layer. *C*) Capsular layer measurements (micrometers) of the adrenals of the *Mrap^−/−^* mice compared with the wild-type mice and in response to lifetime corticosterone replacement. *D*) *Shh* expression in the adrenal gland of male *Mrap^+/+^* (*n* = 6) and *Mrap^−/−^* (*n* = 6) mice and of the animals after lifetime corticosterone replacement (GC) (*Mrap^+/+^*, *n* = 4; *Mrap^−/−^*, *n* = 5) shows an increase in *Shh* expression in *Mrap^−/−^* mice. *E*, *F*) *In situ* hybridization demonstrated expression of *Shh* outside ZG in the adrenals of *Mrap^−/−^* mice (*E*) and in the animals after lifetime corticosterone replacement (GC) (*F*). CX, cortex; M, medulla. Scale bars, 50 µm. One-way ANOVA with Bonferroni multiple comparison testing. *P < 0.05, **P < 0.01, ***P < 0.001. Ns, not significant.

The adrenal capsule of 8-wk-old *Mrap^−/−^* mice was thickened compared with the wild-type littermates in absolute measurement (in micrometers) (**Fig. 4*A***). The capsule thickness was due to an increase in cell number rather than extracellular matrix expansion (Fig. 4*A* and Supplemental Fig. 4*A*). This capsular phenotype is established prior to birth (Supplemental Fig. 3*E*). Studies have demonstrated the importance of the adrenal capsule in the control of adrenocortical cell renewal and zonation (15, 18). More recently, PKA and ACTH signaling have been implicated in the regulation of adrenal zonation determining the ZG/ZF border (23).

To elucidate whether the adrenal capsular phenotype was due to the lack of ACTH signaling or the absence of GCs, we performed corticosterone replacement of *Mrap^−/−^* mice and sex-matched, wild-type littermates after weaning. In this case, drinking water was supplemented with corticosterone starting at E17.5 until 8 wk of age when the phenotype was assessed. With this strategy, which we call “lifetime corticosterone treatment,” corticosterone levels between the wild-type and *Mrap^−/−^* littermates were normalized (Supplemental Fig. 4*B*). Examination of the capsule of *Mrap^−/−^* mice that had received lifetime corticosterone treatment revealed that chronic GC administration partially reduced the thickness of the capsular layer, suggesting that GC depletion partly contributed to the capsular phenotype independent of ACTH signaling (Fig. 4*B*, *C*). The absence of ACTH signaling is likely to further contribute to capsule thickness, as demonstrated by an increase in thickness in corticosterone-treated, wild-type mice, with normalized corticosterone levels and low ACTH (Fig. 4*C*). Furthermore, capsule thickness in *Mrap^−/−^* GC-treated mice remains significantly greater compared with *Mrap^+/+^* GC-treated mice, which would suggest an MRAP-independent effect (Fig. 4*C*).

### *Shh* signaling is altered in *Mrap^−/−^* adrenal glands and is not normalized by treatment with GCs

SHH signaling is one of the key pathways regulating adrenal zonation and identifies progenitors of steroidogenic lineages (18, 21). The expression of *Shh* was quantitatively increased in the adrenal glands of *Mrap^−/−^* mice compared with the wild-type littermates (Fig. 4*D*). *In situ* hybridization demonstrated *Shh*-positive cells throughout the adrenal cortex in *Mrap^−/−^* mice, as opposed to the expected subcapsular localization observed in wild-type adrenals (Fig. 4*E*). These data suggest that loss of MRAP-mediated ACTH signaling and/or GC depletion could be the cause of SHH progenitor accumulation. Lifetime corticosterone replacement did not reverse this phenotype, indicating that the *Shh* pathway is likely to depend on MRAP-mediated ACTH signaling rather than on GC levels (Fig. 4*F*). *Shh* expression measured by qRT-PCR after GC was also statistically unchanged in *Mrap^−/−^* mice. Expression of *Gli1*, a transcriptional target of SHH signaling in the adrenal gland (18), was unaffected in the adrenals of *Mrap^−/−^* mice (Supplemental Fig. 4*C*). The expression of other GLI family members (*Gli2* and *Gli3*) was also unchanged (Supplemental Fig. 4*D*, *E*).

### β-Catenin signaling is dysregulated in the adrenal cortex of *Mrap^−/−^* mice

**Figure 5:**
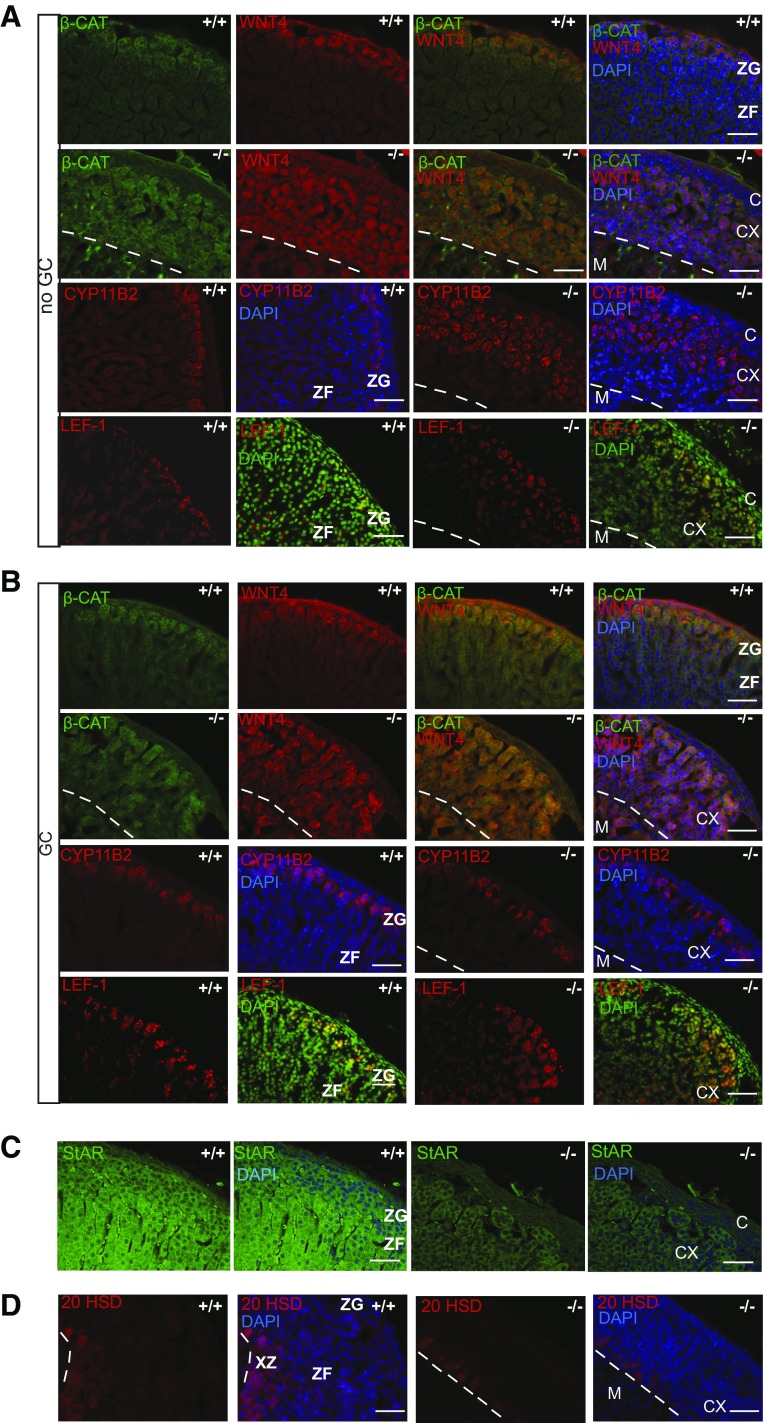
Dysregulation of WNT4/β-catenin signaling in *Mrap^−/−^* mice. *A*) Immunohistochemistry showing localization of β-catenin (β-CAT), WNT4, CYP11B2, and LEF1 in the adrenal glands of *Mrap^+/+^* and *Mrap^−/−^* mice. *B*) Immunohistochemistry showing β-CAT, WNT4, CYP11B2, and LEF1 in response to lifetime corticosterone replacement. Cell nuclei are stained with DAPI as blue, with the exception if LEF1 images where the nuclei are stained green to highlight colocalization of LEF1 with nuclei. CX, cortex; M, medulla. Scale bar, 50 µm. *C*) Immunohistochemistry with anti-StAR antibody shows impairment of steroidogenic potential of the cells in the adrenal cortex of *Mrap*^−/−^ mice. *D*) Immunostaining of the adrenal glands with anti-20αHSD antibody shows staining surrounding the medulla in both wild-type and mutant mice. CX, cortex; M, medulla; XZ, X zone. Dashed line indicates medullary boundary. Scale bars, 50 µm.

Recent studies implicated canonical wnt/β-catenin signaling in adrenal gland zonation (23–25). Therefore, we tested the function of this pathway in the adrenals of *Mrap^−/−^* mice. Immunostaining for WNT4 and β-catenin demonstrated expression in the ZG in wild-type, mice as previously reported (**Fig. 5*A***). *Mrap^−/−^* mice demonstrated WNT4 and β-catenin staining throughout the adrenal cortex in both lifetime corticosterone-replaced and nonreplaced animals (Fig. 5*A*, *B*).

Expression of WNT4 and β-catenin is associated with ZG cell identity (26, 27). Therefore, we examined the expression of CYP11B2 in these cells. It appeared that some of the cells that were positive for WNT4 and β-catenin expression were negative for CYP11B2 and hence were not aldosterone producing (Fig. 5*A* and Supplemental Fig. 4*F*). These cells were also negative for DAB2, another ZG-specific marker (Supplemental Fig. 4*G*). Expression of *Lef1*, a downstream component of the β-catenin pathway, was not affected in *Mrap*^−/−^ mice (Fig. 5*A*). The level of *Lef1* mRNA expression was increased in *Mrap*^−/−^ adrenals after lifetime GC treatment (Supplemental Fig. 4*H*). In wild-type mice, immunohistochemistry showed that LEF1 was localized to the nuclei of the ZG (Fig. 5*A*) as previously reported (28). LEF1-positive cells in *Mrap^−/−^* adrenal glands were also found to be expressed more widely in the cortex compared with wild-type mice, although LEF1-positive cells did not penetrate as deeply into the cortex compared with WNT4 and β-catenin cells (Fig. 5*A*). There was no effect in response to chronic GC administration on LEF1 distribution, suggesting GCs are unlikely to regulate this signaling pathway (Fig. 5*B*).

AXIN-2 is another transcriptional target of the canonical β-catenin pathway. Consistent with previous reports (14), expression of *Axin2* was increased upon chronic GC administration (Supplemental Fig. 4*I*). Similar to *Lef1* expression, *Axin2* levels in untreated *Mrap^−/−^* mice were similar to the wild-type littermates, and mutant mice responded to GC treatment by an increase in *Axin2* expression; however, this response was less prominent compared with that observed in wild-type mice (Fig. 5*A*, *B*). These findings suggest that, although WNT4 and β-catenin are accumulated in the vast majority of cells throughout the cortex of the *Mrap^−/−^* adrenals, their transcription targets LEF1, AXIN-2, and CYP11B2 are activated only in a proportion of these cells. The adrenal cortical cells in the *Mrap^−/−^* mice, including those negative for CYP11B2 and LEF1, showed impairment of the steroidogenic potential as assessed by StAR immunostaining (Fig. 5*C*). The WNT4 and β-catenin–positive but CYP11B2-negative cells were also negative for 20α-hydroxysteroid dehydrogenase (20-αHSD), suggesting that they were not fetal X-zone cells (29) (Fig. 5*D*). Furthermore, lifetime treatment with GCs was capable of normalizing the CYP11B2 phenotype in *Mrap^−/−^* mice without changing the LEF1 or AXIN-2 phenotypes (Fig. 5*A*, *B*).

## DISCUSSION

We previously identified mutations in MRAP as a cause of FGD2. Here we describe the first *Mrap*^−/−^ mouse model of ACTH resistance with isolated GC deficiency, representative of patients with FGD2. This study demonstrates the importance of MRAP in adrenal physiology. Furthermore, we show the importance of MRAP in adrenal progenitor cell differentiation and adrenal cortex zonation. In view of the relative postnatal reduction in adrenal sizes in *Mrap^−/−^* mice, MRAP is likely to also play a role in adrenal gland maintenance and self-renewal.

Patients with MRAP mutations are diagnosed earlier in the neonatal period compared with those with MC2R mutations (13); this correlates with the higher neonatal death rates observed in *Mrap*^−/−^ mice. *Mrap*^−/−^ pups died due to impaired maturation of the lungs, rescued by corticosterone administration, indicating that this phenotype is due to corticosterone depletion rather than a direct role of *Mrap* deficiency. *Mc2r^−/−^* pups born from homozygous parents died before P0.5 due to lung failure, suggesting that fetal or maternal corticosterone is important for lung maturation (30). *Mrap^+/−^* breeders with normal adrenal function and ACTH responsiveness delivered *Mrap*^−/−^ pups with uninflated lungs. This finding highlights the importance of fetal rather than maternal glucocorticoids in prepartum lung maturation. We also observed depleted glycogen stores in prebirth embryos, which would lead to compromised nutritional adaptation, severe hypoglycemia, and neonatal death, as described for *Mc2r^−/−^* mice (22). Furthermore, we were able to rescue neonatal *Mrap*^−/−^ lethality by the administration of corticosterone in drinking water from E19.5 until weaning. After weaning, *Mrap*^−/−^ mice with undetectable corticosterone levels survived.

Adult *Mrap*^−/−^ mice exhibited gross morphologic changes in the adrenal cortex and disturbances of the steroidogenic pathway, with total absence of circulating corticosterone. Despite this severe phenotype, aldosterone levels were similar to those in the wild-type littermates. The lack of StAR staining by immunohistochemisty of *Mrap*^−/−^-derived adrenals may represent the inability of the StAR antibody to detect low levels of StAR, measurable by qRT-PCR, that enable normal aldosterone production in picogram per milliliter concentrations. The adrenal medulla, histologically and functionally, appeared to be unaffected. PMNT, an enzyme responsible for conversion of noradrenaline to adrenaline, is expressed in the medulla of corticosterone-deficient *Mrap^−/−^* mice. This is surprising because the expression of PNMT is known to be dependent on GCs (31, 32). There is suggestion of a reduction in PNMT mRNA levels in *Mrap^−/−^* mice by qRT-PCR, although this was not statistically significant. Furthermore, PNMT activity is clearly adequate for the production of adrenaline in *Mrap^−/−^* mice, which was statistically indifferent to the adrenaline levels in *Mrap*^+/+^ littermates at 8 wk of age. However, our mice (both genotypes) received relatively high doses of GCs between E17.5 until weaning; hence, it is unclear if this is sufficient for medullary PMNT expression in the first instance. The absence of mineralocorticoid and the catecholamine deficiency in *Mrap^−/−^* mice make this model uniquely different from the previously reported *Mc2r^−/−^* mice, which exhibited low aldosterone and catecholamines levels (22).

The capsule of *Mrap^−/−^* adrenals was thickened with increased cell number; a similar change is seen in *Mc2r^−/−^* adrenal glands (22), suggesting that this phenotype is likely due to a lack of ACTH signaling and/or GC production. Our data demonstrate the importance of both GCs and ACTH in capsule thickness. Lifetime GC replacement in *Mrap^−/−^* mice partially normalized the thickened adrenal capsule of *Mrap^−/−^* mice, whereas ACTH deficiency in lifetime GC-treated wild-type animals resulted in an increase in adrenal capsule thickness. Moreover, our data suggest that MRAP may independently affect capsule thickness. The adrenal capsule gives rise to steroidogenic cells and is implicated in the regulation of adrenal cell differentiation and zonation. One of the key pathways regulating this process is SHH signaling (18). King *et al.* (18) demonstrated that removing *Shh* signaling reduced the adrenal capsule thickness to a single layer and suggested that SHH acted either as a capsule cell mitogen/chemoattractant for noncapsule mesenchyme or to maintain capsule progenitors. In keeping with this suggestion, we found that *Mrap^−/−^* mice had up-regulated SHH expression and thickening of the capsule. However, we also found ectopic SHH expression throughout the cortex, which is not rescued by lifetime GC replacement. Despite this failure to rescue, the capsule thickness was reduced with GC treatment. This indicates that SHH up-regulation was likely to be due to MRAP-mediated ACTH resistance and not GC depletion. Furthermore, there was dissociation between SHH signaling and capsule thickness. This dissociation could be explained by the lack of change in *Gli1* expression levels from *Mrap^−/−^* adrenal glands, perhaps pointing to a lack of increase in canonical hedgehog signaling in the cortex of these animals. A thickened capsule is also seen in *FgfrIIIb^−/−^* mice, although in this case *Gli1* expression in the capsule is increased (33). How the FGF, SHH, and ACTH signaling co-ordinates the regulation of capsule thickness and progenitor cell differentiation is subject to future investigations.

Another key signaling pathway implicated in the adrenal gland zonation regulation, the WNT4/β-catenin pathway, was dysregulated in the adrenals of *Mrap^−/−^* mice. The canonical WNT4/β-catenin pathway is a driver of ZG identity and is normally restricted to the subcapsular region, where it is coexpressed with SHH and CYP11B2-positive cells (26, 27, 34). *Mrap^−/−^* mice demonstrated ectopic accumulation of WNT4/β-catenin throughout the cortex, mirroring the SHH expression. This is consistent with the hypothesis that ACTH signaling *via* PKA inhibits the WNT4/β-catenin pathway (15). However, a proportion of such cells were negative for the downstream target of the pathway LEF1, DAB2, and CYP11B2, suggesting that, despite WNT4/β-catenin accumulation, such cells do not contribute to aldosterone production. This suggests that canonical WNT signaling is not active in the inner cortical CYP11B2-negative cells in the adrenal glands of *Mrap^−/−^* mice.

In summary, MRAP deficiency results in isolated GC deficiency, making this a unique model replicating the human FGD phenotype. We showed that the key pathways regulating adrenal zonation were dysregulated in the adrenal glands of *Mrap^−/−^* mice, demonstrating the role of MRAP-mediated ACTH signaling in the regulation of adrenal progenitor cell differentiation (15, 23). Contrary to current views, in this study we show that canonical β-catenin through LEF1 does not always drive ZG identity. Our mouse model may point to noncanonical WNT signaling pathways in adrenal gland zonation. Moreover, GC replacement did not rescue the SHH or WNT4/β-catenin pathway deregulation but decreased the thickness of the adrenal capsule of *Mrap^−/−^* mice, indicating a potential role for long-term GC treatment in regulating the stem cell niche physiology in ACTH resistance syndromes.

## Supplementary Material

This article includes supplemental data. Please visit *http://www.fasebj.org* to obtain this information.

## Abbreviations

ACTH: adrenocorticotropin hormone
FGD: familial glucocorticoid deficiency
GC: glucocorticoid
H&E: hematoxylin and eosin
MC2R: melanocortin 2 receptor
MRAP: melanocortin 2 receptor accessory protein
qRT-PCR: quantitative RT-PCR
SHH: sonic hedgehog
ZF: zona fasciculata
ZG: zona glomerulosa
ZR: zona reticularis
