# Supplementary figures and images for: MRAP deficiency impairs adrenal progenitor cell differentiation and gland zonation
Source: FASEB J. 2018 Jun 7;32(11):6186–96. doi: 10.1096/fj.201701274RR (PMC6181639; doi:10.1096/fj.201701274RR)

Supplemental Fig 1

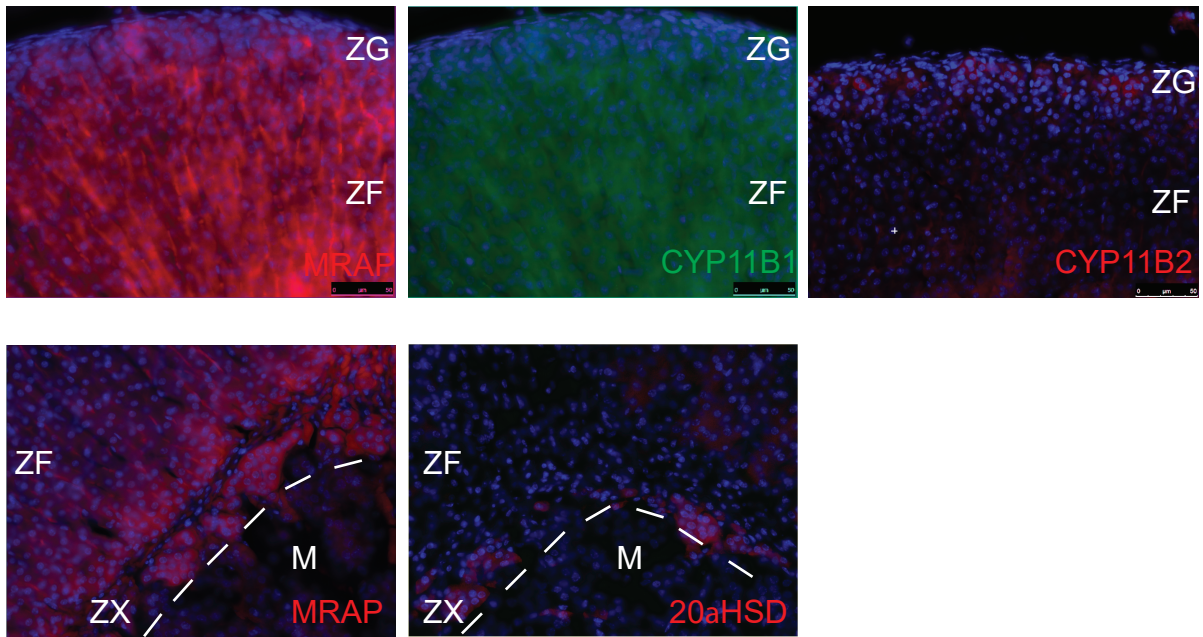

Supplement: Supplementary file 1 [file fj.201701274RR.sf1.pdf]

# Supplemental Fig 2

A

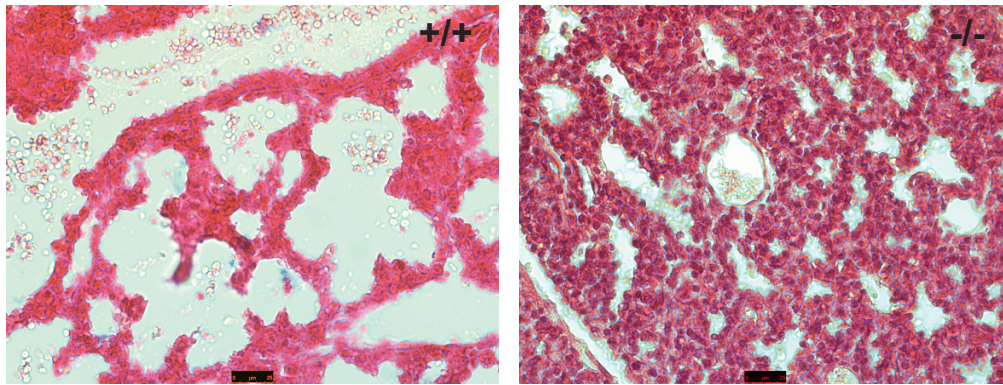

B

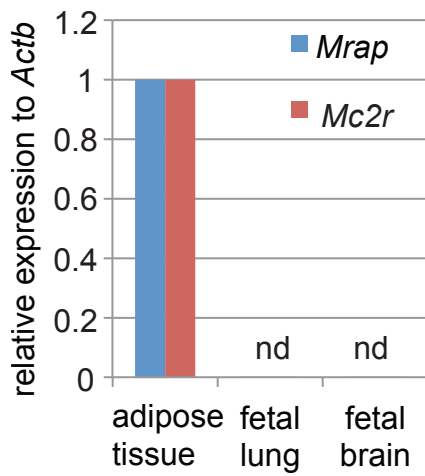

C

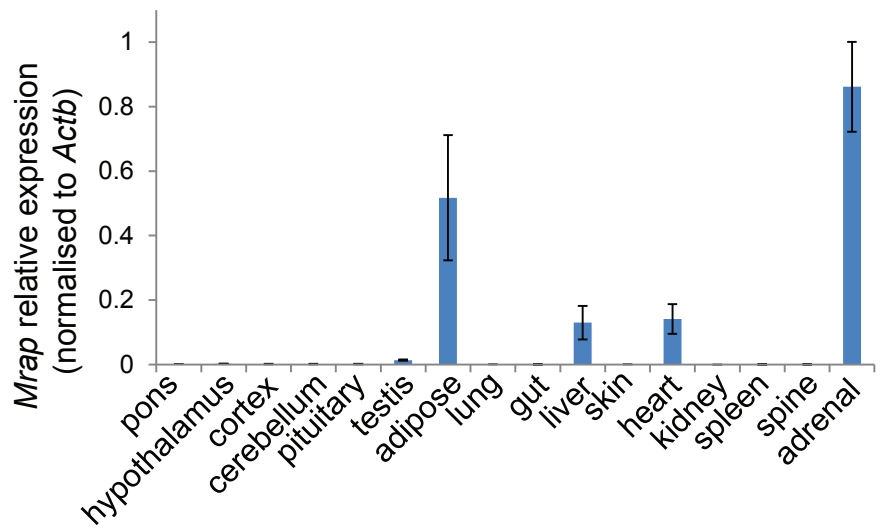

D

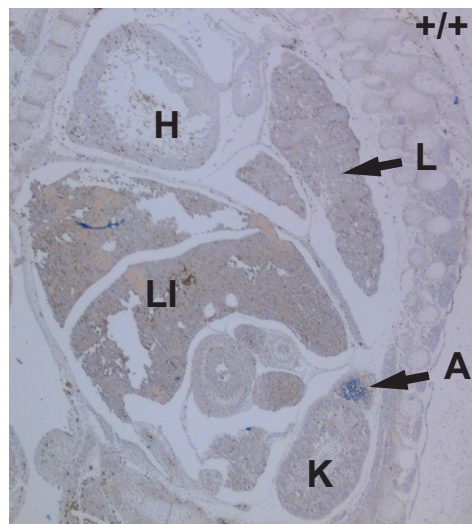

E

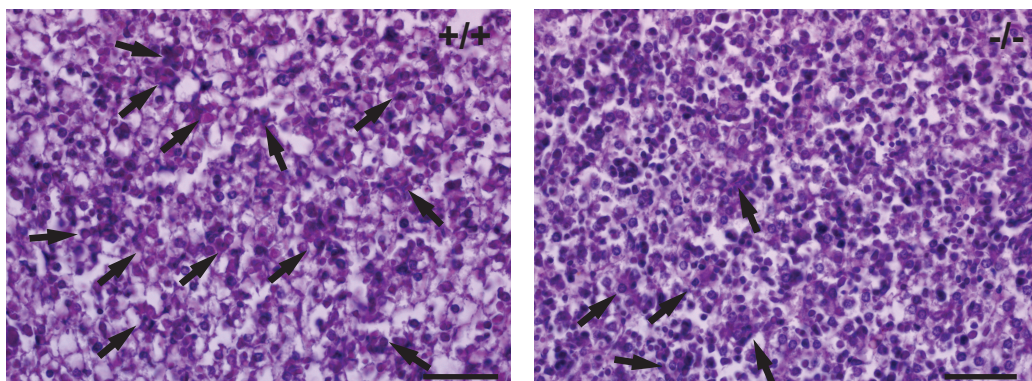

Supplement: Supplementary file 2 [file fj.201701274RR.sf2.pdf]

Supplemental Fig 3

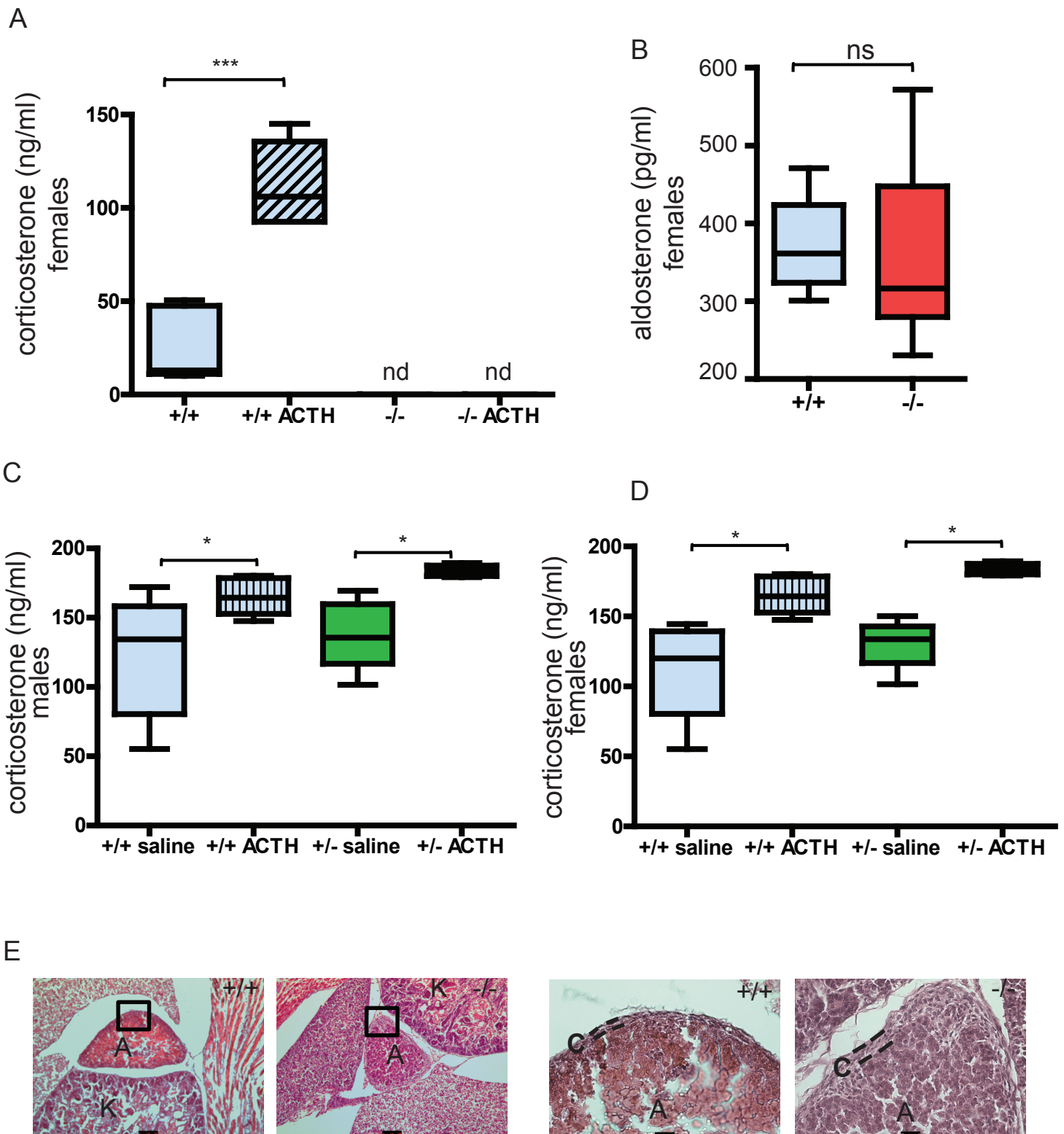

Supplement: Supplementary file 3 [file fj.201701274RR.sf3.pdf]

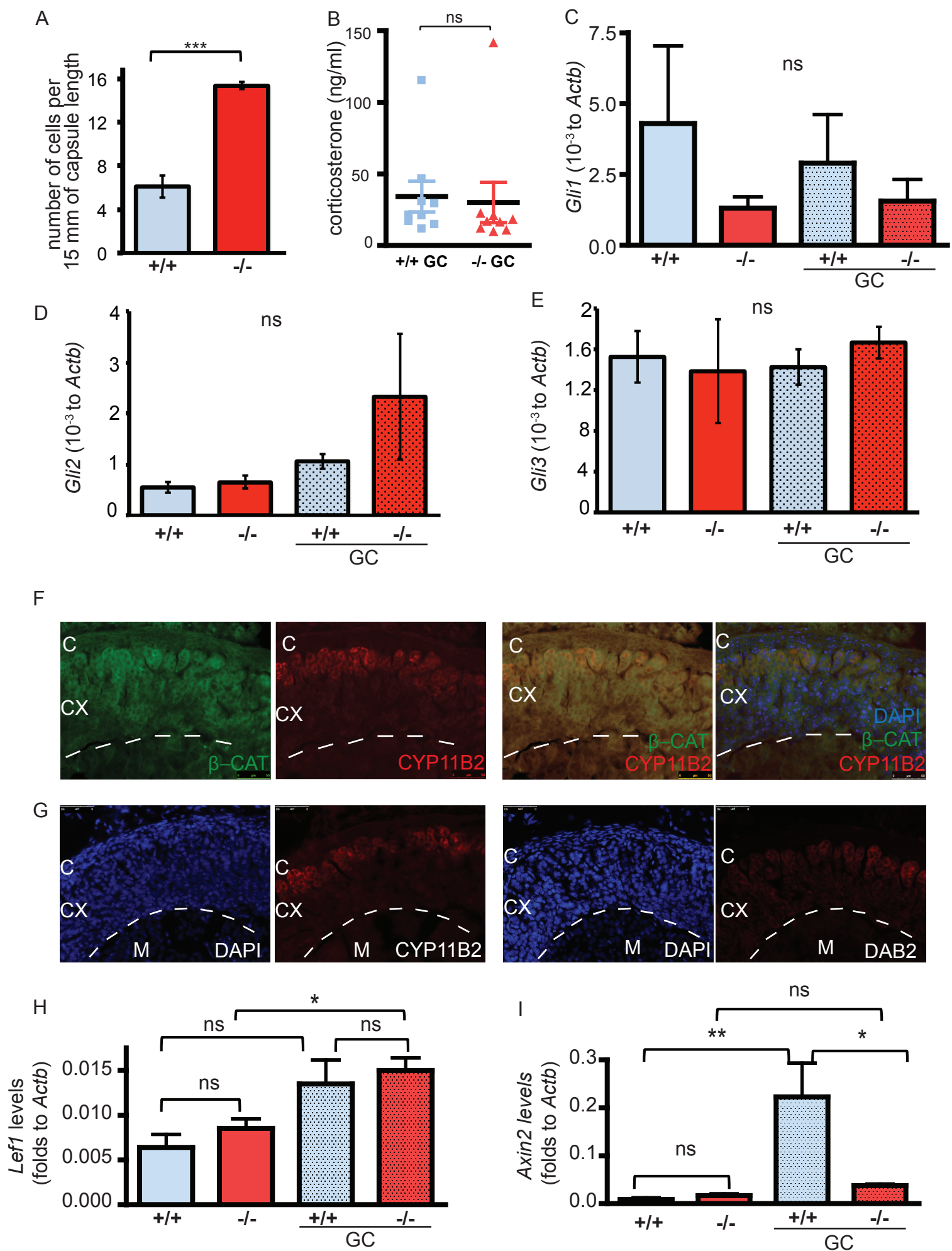

Supplemental Fig 4

Supplement: Supplementary file 4 [file fj.201701274RR.sf4.pdf]
