# Supplementary material for: MRAP deficiency impairs adrenal progenitor cell differentiation and gland zonation
Source: FASEB J. 2018 Jun 7;32(11):6186–96. doi: 10.1096/fj.201701274RR (PMC6181639; doi:10.1096/fj.201701274RR)
Supplement: Supplementary file 5 [file fj.201701274RR.st1.docx]

**Supplemental table 1. TaqMan gene expression assays used in the study**

| **Gene** | **Assay ID** |
| --- | --- |
| *SF-1 (Nr5a1)* | Mm00446826_m1 |
| *Mrap* | Mm00547149_m1 |
| *Mrap2* | Mm01169300_m1 |
| *Cyp11b1* | Mm01204952_m1 |
| *Cyp11b2* | Mm01204955_g1 |
| *Mc2r* | Mm00434865_s1 |
| *Cyp11a1* | Mm00490735_m1 |
| *Hsd3b1* | Mm01261921_mH |
| *Star* | Mm00441558_m1 |
| *At1r (Agtr1b)* | Mm02620758_s1 |
| *Pnmt* | Mm00476993_m1 |
| *Shh* | Mm00436528_m1 |
| *Gli1* | Mm00494654_m1 |
| *Gli2* | Mm01293117_m1 |
| *Gli3* | Mm00492337_m1 |
| *Axin-2* | Mm00443610_m1 |
| *Lef-1* | Mm00550265_m1 |
