# Supplementary material for: MRAP deficiency impairs adrenal progenitor cell differentiation and gland zonation
Source: FASEB J. 2018 Jun 7;32(11):6186–96. doi: 10.1096/fj.201701274RR (PMC6181639; doi:10.1096/fj.201701274RR)
Supplement: Supplementary file 6 [file fj.201701274RR.sd1.docx]

**Supplemental figure legends**

**Supplemental Fig. 1.** Immunohistochemistry with anti-MRAP staining compared with CYP11B1 expression, and MRAP staining compared with 20-HSD in adjacent sections from *Mrap^+/+^* young virgin female adrenals, DAPI shown in blue. Scale bars are 50μm.

**Supplemental Fig. 2.** *(A)* Morphology of the *Mrap^+/+^* and *Mrap^-/-^* E19.5 embryonic lungs as shown by H&E staining suggested glandular-like immature lung structure. The scale bars are 20 µm. *(B)* RT-qPCR analysis of *Mrap* and *Mc2r* RNA expression, normalised to *Actin b* (*Actb*) in the lungs of E15.5 *Mrap^+/+^* embryos (n=3). *(C)* RT-qPCR analysis of *Mrap* RNA expression across different mouse tissue (males; n=3), normalised to *Actin b* (*Actb*). *(D)* *Mrap* in situ hybridisation of E14.5 *Mrap^+/+^* embryo showing *Mrap* expression in the adrenal gland (indicated with arrow) but not in the lungs (L). H-heart; LI –liver; A-adrenal gland; K-kidney. The scale bars are 200 µm *(E)* Periodic acid–Schiff staining for glycogen of *Mrap^+/+^* and *Mrap^-/-^* E19.5 embryonic liver shows reduction in the glycogen store (magenta, indicated with arrows). The scale bars are 50 µm.

**Supplemental Fig. 3.** *(A)* Plasma corticosterone levels (ng/ml) at the basal level and in response to ACTH in the female *Mrap^+/+^* (n=4) and *Mrap^-/-^* (n=7) mice *(B)* Comparison of plasma aldosterone levels (pg/ml) between female mice *Mrap^+/+^* (n=4) and *Mrap^-/-^* (n=7) mice *(C)* Plasma corticosterone levels (ng/ml) in response to saline or ACTH in the male *Mrap^+/+^* (n=6) and *Mrap^+/-^* (n=6) mice and *(D)* female *Mrap^+/+^* (n=6) and *Mrap^+/-^* (n=6) mice *(E)* Capsular layer is increased in the E19.5 *Mrap^-/-^* embryos as shown by H&E staining of the adrenal gland. The black boxes indicate the area of higher magnification. The black bars highlight the capsule thickness; A – adrenal gland; C-capsule; K-kidney; the scale bars top panel 100m; bottom panel 25m.

**Supplemental Fig. 4.** *(A)* Cell number in the adrenal capsule of the *Mrap^-/-^* (n=3) and *Mrap^+/+^* (n=3) mice. Data represents mean cell number per 15um of capsule length +/- SD *(B)* Plasma corticosterone levels (ng/ml) of *Mrap^+/+^* (n=5) and *Mrap^-/-^* (n=6) without life-time corticosterone replacement and from mice with lifetime corticosterone treatment (*Mrap^+/+^* GC n=5; *Mrap^-/-^* GC n=5) assessed at the age of 8 weeks. *(C)* *Gli1* *(D) Gli2* *(E)* *Gli3* RNA expression levels in adrenal glands of *Mrap^+/+^* and *Mrap^-/-^* mice, n6 in each group (F) Co-staining of -catenin and CYP11B2 in *Mrap^-/-^* mice adrenals (G) Immunohistochemistry with anti-DAB2 staining compared with CYP11B2 expression in adjacent sections from *Mrap^-/-^* mice adrenals, DAPI shown in blue. Scale bars are 50μm (H) *Lef-1* and (I) *Axin 2* RNA expression levels in adrenal glands of *Mrap^+/+^* (n=6) and *Mrap^-/-^* (n=6) mice and of the animals after lifetime corticosterone replacement (GC) (*Mrap^+/+^* n=4; *Mrap^-/-^* n=5), normalised to actin b (*Actb*).
